# Multi-Layer Fabric Composites Combined with Non-Newtonian Shear Thickening in Ballistic Protection—Hybrid Numerical Methods and Ballistic Tests

**DOI:** 10.3390/polym15173584

**Published:** 2023-08-29

**Authors:** Maciej Roszak, Dariusz Pyka, Mirosław Bocian, Narcis Barsan, Egidijus Dragašius, Krzysztof Jamroziak

**Affiliations:** 1 Department of Mechanics, Materials Science and Biomedical Engineering, Wrocław University of Science and Technology, Smoluchowskiego 25, 50-370 Wrocław, Poland; dariusz.pyka@pwr.edu.pl (D.P.); miroslaw.bocian@pwr.edu.pl (M.B.); krzysztof.jamroziak@pwr.edu.pl (K.J.); 2 Department of Environmental Engineering and Mechanical Engineering, Vasile Alecsandri University of Bacau, Calea Marasesti 157, 600115 Bacau, Romania; narcis.barsan@ub.ro; 3 Department of Manufacturing Engineering, Faculty of Mechanical Engineering and Design, Kaunas University of Technology, Studentu Str. 56, LT-51424 Kaunas, Lithuania; egidijus.dragasius@ktu.lt

**Keywords:** ballistics, impact loads, multi-layer armor, simulations, fabric shields, homogenization method, non-Newtonian fluid

## Abstract

Multi-layer fabrics are commonly used in ballistics shields with a lower bulletproof class to protect against pistol and revolver bullets. In order to additionally limit the dynamic deflection of the samples, layers reinforced with additional materials, including non-Newtonian fluids compacted by shear, are additionally used. Performing a wide range of tests in each case can be very problematic; therefore, there are many calculation methods that allow, with better or worse results, mapping of the behavior of the material in the case of impact loads. The search for simplified methods is very important in order to simplify the complexity of numerical fabric models while maintaining the accuracy of the results obtained. In this article, multi-layer composites were tested. Two samples were included in the elements subjected to shelling. In the first sample, the outer layers consisted of aramid fabrics in a laminate with a thermoplastic polymer matrix. The middle layer contained a non-Newtonian shear-thickening fluid enclosed in hexagonal (honeycomb) cells. The fluid was produced using polypropylene glycol and colloidal silica powder with a diameter of 14 µm in the proportions of 60/40. The backing plate was made using a 12-layer composite made of Twaron^®^ para-aramid fabrics with a DCPD matrix—not yet used in a wide range of ballistics. Then, numerical simulations were carried out in the Abaqus/Explicit dynamic analysis. The Johnson–Cook constitutive strength model was used to describe the behavior of elastic–plastic materials constituting the elements of the projectiles. For the non-Newtonian fluid, a Up-Us EOS was used. The inner layers of the fabric were treated as an orthotropic material. Complete homogenization of the sample layers was carried out, thanks to which each layer was treated as a homogeneous continuum. As a parameter of fracture mechanics for shield components, the strain criterion was used with the smooth particles hydrodynamics method (SPH). Then, the results of simulations were compared with the results of the ballistic test for both samples placed next to each other, which resulted in the formation of a multi-layer composite in one ballistic test subjected to impact loads during firing with a 9 × 19 mm Parabellum FMJ projectile with an initial velocity of 370 ± 10 m/s. The results of numerical tests are very similar to the ballistic tests, which indicates the correct mapping of the process and the correct conduct of layer homogenization. The applied proportions of the components in the non-Newtonian fluid allowed a reduction in the deflection compared to previous studies. Additionally, the proposal to use a DCPD matrix allowed to obtain a much lower deflection value compared to other materials, which is a novelty in the field of production of ballistic shields.

## 1. Introduction

Aramid fibers are still the main material used for the production of multi-layer composites for ballistic shields, especially in the case of protection against pistols or revolvers. Aramid fibers in ballistic shields are used in a special form of densely woven fabrics. Many variations of the starting materials are used such as Kevlar 29, Kevlar 49, Kevlar HT, Twaron CT, etc. Aramid fibers are characterized as non-flammable, thermally stable and with a high index of ballistic protection. They are used, as mentioned earlier, in light shields but also as a backing material in hard ballistic inserts or helmets [1,2]. Fibrous materials are used not only in the form of multi-layer fabrics but also in many cases polymer matrices are additionally used. The literature indicates that the impact of the matrix, which has in many cases significantly worse mechanical properties, is not so trivial. Under impact, loads in layered composites will be associated with the deformation of the ballistic shield and the striking projectile, and then in the rupture of fibrous layers. As a result of exceeding the strength limit, fiber shearing, delamination and matrix cracking can be observed, followed by heat generation caused by friction between the projectile and the composite. This process greatly depends on the mechanical properties of the fibers. Aramid fibers become more elastic under the impact load; therefore with this dominant element causing dissipation of impact energy, there will be delamination in the matrix until its rupture. It is estimated that in such cases the energy absorber in the process of brittle fracture of the matrix may even reach 30–35% [1,3,4].

The search for optimal models of the behavior of fabrics and ballistic laminates can be successfully carried out through computer simulation programs. Performing dynamic phenomena requires precision and the use of appropriate modeling methods to obtain results close to reality. There are many ways to define and numerically analyze fibrous materials in the literature. These methods of modeling composites mainly differ in two types: modeling at the level of yarns or layers. The first and very common way is to model composites using the RVE method (representative volume elements). This method allows modeling of a small piece of fabric taking into account the interlacing of the fabric and the matrix. Then these cells are duplicated throughout the volume of the material. The theory behind the application of RVE is extensively described in the source [5]. Models of this type reproduce well the behavior of the material in its entire volume and are much more computationally efficient than those modeled at the level of a single fiber using, for example, 3d elements [6,7,8]. In another approach, there is a possibility to treat the whole composite or individual layers as an idealized continuum by membrane, 3d volume or shell elements [9,10,11]. This is the simplest method to simulate composite behavior and the least computationally demanding. However, this way does not consider the behavior of a single fiber as it is based on great simplifications. Additionally, in the case of matrix-containing composites, their influence on the behavior of the material may be neglected. Also, it does not take into account factors such as interactions between fibers, fiber movement in the plane, fiber untangling and fiber–bullet interaction, which are the dominant factors in the energy absorption in textile armors [12,13]. The next way is to model composites at the fiber level by articulated bar, shell or beam elements [14,15,16]. These methods reflect the behavior of the material much better because they take into account factors such as interlacing of fibers, friction at the fiber level, etc. Pin-jointed bars are the least computationally demanding method compared to others, at the yarn level [17,18]. The truss models are more simple and capable but fail to incorporate the interaction between different fabric layers, which is important, especially in multi-layer composites. Numerical models developed with the use of 3D solid elements, taking into account the real geometry of the weave and the effect of friction, are more accurate, but require high computational effort [19,20,21]. Using yarn-level methods for multi-layer, larger-scale analysis can be incredibly computationally demanding. In many cases, the influence of the matrix is also omitted when modeling at the fiber level. The last ways to model composites are mixed methods (hybrid elements method) and homogenization methods. The first allows modeling of the fibers in a limited range—closest to the point of impact of the bullet, and in further areas, the material arrangement is treated as a continuum. This allows for mapping the behavior of fibers (friction between fibers, interlacing) while reducing computational requirements by treating the fabric to a greater extent as a continuum [22,23,24]. In the second method, however, there is the possibility of homogenization at a selected level, thanks to the use of the law of mixtures, or using simplifications for a single RVE, mapping the effect of both fibers and the matrix [25,26,27]. As the authors indicate [2,22], depending on the expected results, the use of simplified methods to map the behavior of fabric composites is not wrong, because sometimes the most important factor tested during the simulation is, for example, deflection or failure and not specifically a yarn–yarn interaction.

Each method requires a specific method of fiber-matrix modeling. In addition, as already mentioned, the modeling of polymer matrix composites should be taken into account. For the best reproduction of the material, no factor can be overlooked, especially the matrix, which absorbs energy to a large extent. Due to the complexity of many of the previously mentioned methods, the search for simplified methods for a good representation of the behavior of composite materials is very important. The development of the homogenization method allows the modeling of composites, using the law of mixtures, which allows for the simplifications of the numerical model, while taking into account the matrix influence, and the obtained results allow for a good mapping of the material behavior [28].

Currently, there are many methods to improve the energy absorption capacity of multi-layer composites, not only by using modern materials as the matrix but also by improving the material with non-Newtonian fluids [2,29,30]. Unlike a Newtonian fluid, the viscosity of a non-Newtonian fluid is not constant under isobaric conditions but varies with time. The flow curve (plot of shear stress versus shear rate) of such a fluid is not a linear function. There are many types of non-Newtonian fluids; however, in the case of ballistic shields, the most desirable property of a rheological fluid is its hardening with increasing load. As a result, the usefulness of this type of material is practically limited to shear-thickening liquids. As a result, the material is hardened upon impact. Shear-thickened fluids are intelligent (materials capable of changing their properties, shape, etc. under the influence of external factors) materials that are non-Newtonian in nature and exhibit solid-like properties when the shear force or shear rate exceeds the critical shear stress or critical shear rate, resulting in a rapid increase in viscosity [31,32]. Due to this unique shear-thickening rheological property, they facilitate the absorption of impact energy for protection in the event of an impact. These materials can come in two forms. Firstly, as impregnations that are used directly on fabrics [2,33,34]. In the second case, however, as a liquid directly enclosed in cells isolated from fabrics [35,36]. During the experimental research contained in the sources [37,38], the authors focused on the analysis of the impact of impregnation of Kevlar layers with STF fluids. As a result of impregnation, the composites had much better parameters in terms of puncture resistance, as well as much better dissipation of bullet impact energy. It is considered that the better performance of the ballistic shield is due to the increase in friction between the yarns due to the deposition of nanoparticles on the yarn/filament surface. In turn, in other studies [39,40], the influence of the size and the percentage content of particles in the carrier liquid was examined, which is useful in the case of composites where the non-Newtonian fluid is isolated from the fabrics in the form of closed cells. In this case, too, a much higher puncture resistance and deflection reduction is achieved. It is believed to be due to this viscous energy dissipation due to the higher viscosity of STF. 

The aim of this work is to carry out a complete homogenization of the layers of the multi-layer composite consisting of homogenizing the mechanical properties in the entire volume of individual layers. The use of simplified simulation methods is extremely important from the point of view of the complexity and computational cost of numerical models. In a previous paper, the authors presented the possibility of using homogenization at the volume level of the entire composite level, layer and fabric/matrix layer, using volumetric elements, and the results showed that, the layer-based modeling works best, but the possibility of homogenization at the level of layers consisting of non-Newtonian fluid has not been tested so far [41]. In addition, an important aspect to which the authors have devoted attention is the limitation of injuries that may occur during dynamic deflections during the shelling of fabric composites. Minimization of the BFS range is an important parameter when assessing behind-armor blunt trauma (BABT). In this work, an increased proportion of silica particles in the composition of the non-Newtonian fluid was used based on the previous investigations [42].

## 2. Materials and Methods

The experimental part includes the preparation of multi-layer samples of para-aramid fabrics on a modified DCPD (dicyclopentadiene) matrix and a non-Newtonian shear-thickening fluid enclosed in hexagonal (honeycomb) cells. After preparation, the samples were subjected to ballistic tests under 9 × 19 mm Parabellum FMJ (full metal jacket) ammunition with an initial velocity of 370 ± 10 m/s. Then, a fragment of the sample was cut out and examined using SEM to estimate the percentage composition of the fibers and the matrix mixtures to carry a homogenization of layers of samples composed of fibers on a DCPD matrix. A fragment of a multi-layer composite consisting of hexagonal cells of a honeycomb structure, in which a non-Newtonian fluid was enclosed, was also homogenized and treated as a homogeneous continuum. After that, numerical tests were carried out, during which the adopted method of modeling with layer homogenization was verified.

### 2.1. Sample Preparation

The backing plate laminate samples consisted of two components: epoxy matrix and the Twaron^®^ para-aramid (Teijin Aramid, Arnhem, The Netherlands) fabric of 280 g/m^2^ basic weight. The fabric thickness of 0.4 mm has a fiber density of 1.44 g/cm^3^ and a fiber diameter of 12 µm. The fabric had a plain weave structure. For laminate preparation, 12 layers of fabric were used, which resulted in a sample thickness of 4.4 mm and a weight of 224 g, other dimensions were 130 × 130 mm. Modified DCPD (dicyclopentadiene) was used as the matrix resin, which is a by-product secreted from processing crude oil. The polymerization process was carried out in the presence of a second-generation Grubbs catalyst. The paper matrix applied in this is characterized by improved parameters of static tensile and bending strength, high relative elongation at rupture and high compressive strength in comparison to other types of resins. After super-saturation with DCPD, the material was subjected to pressure at room temperature (21 °C) at a pressure of approx. 1.5 MPa and was left for 12 h. The outer layers of the sample consisting of outer fabric layers and containing the non-Newtonian fluid were made using the same technique as the previously described sample, but a different material was used. To prepare a DCPD matrix laminate outer layer of the sample, Twaron^®^ (Teijin Aramid, Arnhem, Netherlands) aramid fabric of 173 g/m^2^ basic weight and plain weave structure was used as reinforcement. For laminate production, 4 layers and 2 layers of fabric were used, which resulted in a layer thickness of 4 mm and 2 mm, respectively. The liquid sample (STF) with dimensions of 110 × 100 mm, with hexagons with a diagonal of 10 mm and a height of 12 mm. The non-Newtonian liquid was 100 g of propylene glycol per 70 g of silica particles. This translated into 40% of silica with 60% of propylene glycol. Silica powder was added to the glycol used as the base of the non-Newtonian liquid. Silica or silicon dioxide, with the formula SiO_2_, is an inorganic chemical compound. Making samples consisted of accurately measuring the appropriate amounts of each of the components. The measured amount depended on the desired percentage of them in a given sample. After preliminary manual mixing, the sample materials were sent to a high-powered mixer. Mechanically assisted mixing was necessary due to the resistance of the liquid during mixing. As the proportion of silica increased, it became more and more difficult to obtain an appropriate degree of combining the components. As a result of intensive mixing, a significant amount of air was introduced into the non-Newtonian fluid. Its bubbles strongly affect the test results by lowering the impact resistance. The liquid gassed in this way was sent to the autoclave, where, under reduced pressure and increased temperature, significant amounts of gas bubbles were removed from the material. The temperature required strict control so as not to boil any of the ingredients. After removal from the autoclave, still-warm containers with samples were additionally placed on a vibrating plate for further removal of bubbles. The table below (Table 1) shows the properties of ingredients used to prepare non-Newtonian shear-thickening fluid.

### 2.2. Ballistic Tests

**Figure 1:**
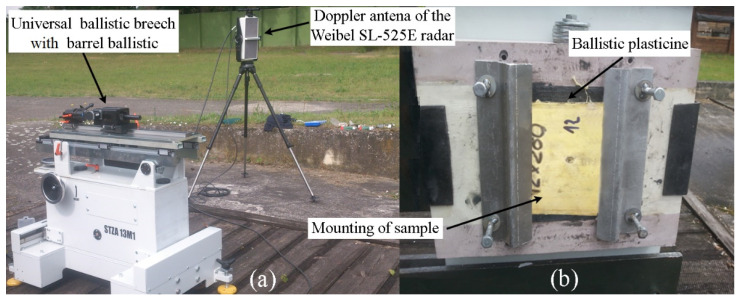
Ballistic stand: (**a**) universal ballistic breech with the 9 × 19 mm barrel ballistic along with the measuring radar; and (**b**) mounting frame with plasticine backing for ballistic targets.

The samples were subjected to impact loads during ballistic tests, using 9 × 19 mm Parabellum FMJ (full metal jacket) pistol ammunition with an initial velocity of 370 ± 10 m/s. The test was carried out in accordance with the PN-EN 1522 standard. A diagram of the ballistic station is shown below (Figure 1). The initial velocity of the projectile was measured using a Doppler antenna of the Weibel SL-525E (Alleroed, Denmark). The firing was carried out with a universal ballistic breech with a ballistic barrel. The primer for assessing the deflection of elastic ballistic plasticine, where subjected, must be applied by switching off the steel carriage three times (a cylinder with a diameter of Φ44 mm and with a spherical notification), weighing 1 kg from a height of 2 m. In accordance with the Polish standard PN-V-87000:2011 practice recess 25 ± 3 mm. Atmospheric conditions: temperature 25 °C, windless, cloudy.

### 2.3. Numerical Analysis

**Figure 2:**
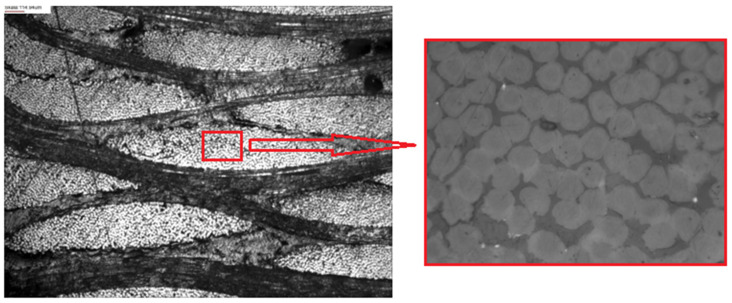
Microstructure of composite visible under a scanning microscope. In the picture on the left, a fragment is marked, which is then zoomed in to determine the histogram.

During the preparation of the models, the homogenization method was used. The use of the homogenization method is justified in the case of fabric composites with a matrix that has a significant impact on the dissipation of kinetic energy during impact, which was described in more detail in the introduction. As was mentioned previously, simulations of fibrous composites require significant computational costs in the case of samples similar to those tested in this article. Therefore, in order to homogenize and simplify the structure of individual layers of the composite, the law of mixtures was applied. In order to estimate the composition of the percentage of fibers and the polymer matrix, a fragment of the sample was cut and analyzed on a scanning microscope. The images obtained as a result of the observation of the microstructure are shown below (Figure 2). During microscopic image observations, it can be seen that the areas between the fibers are filled with the matrix material.

**Figure 3:**
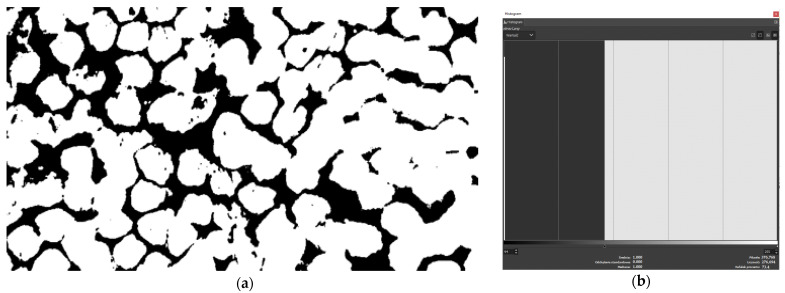
Analysis of histograms using GIMP: (**a**) the final effect of modifying brightness and contrast (fibers—white, matrix—black); (**b**) histogram of obtained colors.

The calculations of the percentage of fibers in the matrix were performed in the GIMP program. For that, 10 areas were selected randomly—such as can be seen above (Figure 2). Calculation of the percentage contained determines the histogram of the percentage of colors in the selected area of the photo. Below (Figure 3) is an example histogram of the percentage distribution of colors in one of the randomly selected areas. Initially, the brightness was changed to separate the fibers and the matrix, then the contrast was maximized to obtain the percentage composition of individual components.

After repeating the aforementioned activity 10 times, the volume fraction of fibers in the composite was estimated at 78.9%. Observing the image above (Figure 2), it can be seen that the individual layers of the composite fabric are densely packed. It was assumed that the percentage of para-aramid fibers for the single yarn is the same as the para-aramid yarn volume in the entire volume of the composite. Also, the value of 78.9% was taken for both the combined fibers in the first and second samples.

**Figure 4:**
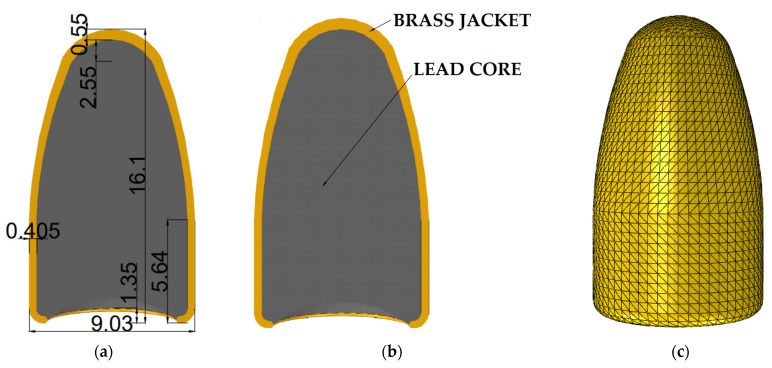
Projectile numerical model: (**a**) geometry; (**b**) components of projectile; and (**c**) mesh.

After determining the percentage of fibers in the matrix, the projectile modeling process was started. The dimensions of the projectile were adopted from the literature [30,44] and can be seen below (Figure 4a). The geometry was mapped in Inventor 2023 Professional. Then, the projectile was imported into the Abaqus calculation program. The projectile was modeled in accordance with a real object and so it contains two components, a jacket made of brass and a lead core (Figure 4b). After the modeling process was completed, the projectile jacket was discretized with tetra elements with an element size of 0.5 mm (Figure 4c).

For metallic components of 9 × 19 mm projectile, the constitutive Johnson–Cook (J-C) strength model has been used. It is one of the most important currently used equations representing the metallic strength of the material model, which is a special type of Mises plasticity model, with analytical forms of the law of hardening and softening of the material under the influence of temperature. The constitutive equation J-C is suitable for computational purposes, thanks to its simplicity and the use of parameters that are relatively easy to determine. It is commonly used in CAE programs, such as Abaqus or LS-Dyna. The formula takes into account the influence of strain, strain rate and temperature on the yield stress value [45,46,47]. This relationship is described by the Equations (1)–(3) below.

(1) $$\sigma_{y}=(A+B{\bar{\varepsilon }}^{p^{n}})(1+Cln{\dot{\varepsilon }}^{*})(1-T^{*m})$$

(2) $${\dot{\varepsilon }}^{*}=\frac{{\dot{\bar{\varepsilon }}}^{p}}{{\dot{\varepsilon }}_{0}}$$

(3) $$T^{*}=\frac{T-T_{room}}{T_{melt}-T_{room}}$$

where: *A*—yield strength, *B*—strengthening constant, *C*—strain rate constant, *n*—strengthening exponent, *m*—thermal softening coefficient, ${\bar{\varepsilon }}^{p}$—effective plastic strain,$\;{\dot{\varepsilon }}^{*}$—effective strain rate (dimensionless), ${\dot{\bar{\varepsilon }}}^{p}$—strain rate, ${\dot{\varepsilon }}_{0}$—reference value for strain rate, T*—homologated temperature (dimensionless), *T_room_*—room temperature, *T_melt_*—melting point, and *T*—current temperature.

Material parameters for the projectile were adopted on the basis of the literature [48] and are summarized in the table below (Table 2).

In the case of shield material, there are many approaches to material definition when it comes to fiber composites. In the first approach, when modeling the layers they are treated as an orthotropic material [9,10,11]. In the next approach, the fiber is treated as an orthotropic element [14,20,49]. In the first place, the mathematical foundations in the case of material orthotropy should be characterized as follows (Equation (4)) [45].

(4) $$\left\{\begin{array}{c} \varepsilon_{11}\\ \begin{array}{c} \varepsilon_{22}\\ \varepsilon_{33}\\ \begin{array}{c} \gamma_{12}\\ \gamma_{13} \end{array} \end{array}\\ \gamma_{23} \end{array}\right\}=\left[\begin{array}{cccccc} \frac{1}{E_{1}} & \frac{-v_{21}}{E_{2}} & \frac{-v_{31}}{E_{3}} & 0 & 0 & 0\\ \frac{-v_{12}}{E_{1}} & \frac{1}{E_{2}} & \frac{-v_{32}}{E_{3}} & 0 & 0 & 0\\ \frac{-v_{13}}{E_{1}} & \frac{-v_{23}}{E_{2}} & \frac{1}{E_{3}} & 0 & 0 & 0\\ 0 & 0 & 0 & \frac{1}{G_{12}} & 0 & 0\\ 0 & 0 & 0 & 0 & \frac{1}{G_{13}} & 0\\ 0 & 0 & 0 & 0 & 0 & \frac{1}{G_{23}} \end{array}\right]\left\{\begin{array}{c} \sigma_{11}\\ \begin{array}{c} \sigma_{22}\\ \sigma_{33}\\ \begin{array}{c} \sigma_{12}\\ \sigma_{13} \end{array} \end{array}\\ \sigma_{23} \end{array}\right\}$$

As can be seen from Equation (1) above, an orthotropic material needs 9 constants to be properly defined. It is possible to use simplifications that allow to reduce the number of parameters to be entered. In both cases (when defining the entire layer as an orthotropic material and when treating the fiber as orthotropic), the material can be defined as transversely isotropic. For the first approach, in the case of weave fabrics in which the directions of interlacing coincide with the direction of the main axes of the adopted coordinate system the following relationships between material parameters can be obtained (5).

(5) $$E_{1}=E_{2}$$

(6) $$v_{13}=v_{23}\;$$

(7) $$G_{13}=G_{23}$$

In the second approach, the transverse isotropy allows for the following simplifications (6). This is caused by the fact that in this case, the adopted coordinate system allows to cover one axis with the direction of the fiber. The other axes of the coordinate system are perpendicular to the direction of the fiber.

(8) $$E_{2}=E_{3}$$

(9) $$v_{12}=v_{13}\;$$

(10) $$G_{12}=G_{13}$$

In the method of homogenization and mixed homogenization, the law of mixtures is applied, so in this case both the influence of the fabric and the matrix are taken into account, which allows taking both factors on the strength of the composite in a more simplified way, but still taking matrix influence into account, which is essential in composites containing matrix. The calculation of the values of individual parameters can be obtained by multiplying the individual parameters with their percentage share [26,28,29]. The law of mixtures makes it possible to consider a composite as a material bi-component fabric with a unidirectional fiber arrangement. It can be also used for a three-dimensional approach. Properties (density, Young’s modulus, Poisson’s ratio, etc.) can be described as follows (7):

(11) $$P_{comp}=\left(P_{1}V_{1}+\cdots +P_{n}V_{n}\right)$$

where: *P_comp_*—properties of composite, and *V*—volume.

Initially, the data used to perform the homogenization are shown in the table below (Table 3). Then, the homogenization of the fabric and matrix material was carried out. The percentage share of individual components was previously calculated on the basis of histograms, as presented at the beginning of this subchapter. 

After applying the rule of mixtures, the final properties used during simulations can be seen below (Table 4).

For modeling non-Newtonian fluids, many techniques can be found. These models are based on analytical shock wave propagation models for dense suspension and on models of unsteady wave propagation. Both approaches of this type to the subject of modeling have been extensively described in the source [31]. The analytical foundations of steady-state propagation are closely related to the state of aggregation of the material, the shape of the reinforcing particles and the percentage of phases. These frames form the basis of what is expected from the experimental Hugoniot shock in the event of thick slurries that thicken as a result of compression at high strain rates [52,53]. This analysis indicates that in areas of low velocity and low pressure of the Hugoniot suspension, wave propagation in the mixture will be dominated by the compressibility of the liquid suspending phase. Furthermore, in the literature the relationship between the volume fraction of a suspension and the shock Hugoniot has been demonstrated, indicating that any increase in the volume fraction of the suspension should be coupled to an increase in the shock wave propagation velocity of the mixture [31]. For the non-Newtonian fluid, the following material data were adopted based on the literature data [2,31] and are presented in the table below (Table 5).

**Figure 5:**
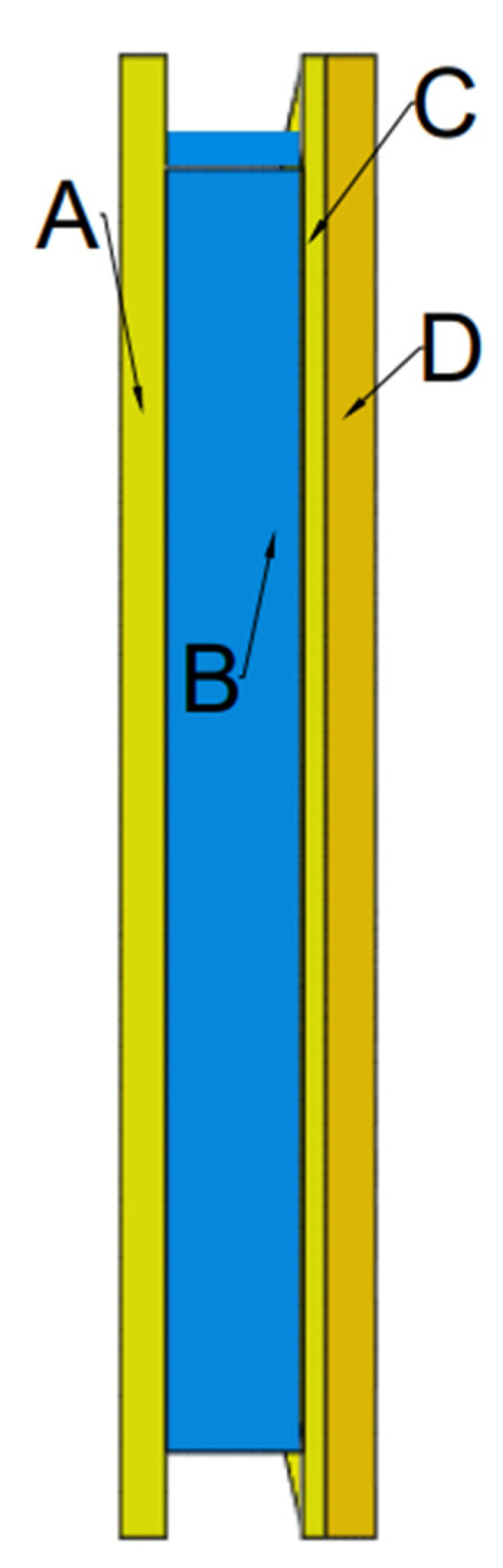
Numerical model of sample: A—4 layers of 173 g/m^2^ Twaron^®^ on modified DCPD matrix with a total thickness of 4 mm; B—non-Newtonian fluid in a honeycomb cells with a total thickness of 12 mm; C—2 layers of 173 g/m^2^ Twaron^®^ on modified DCPD matrix with a total thickness of 2 mm; and D—12 layers of 280 g/m^2^ Twaron^®^ on modified DCPD matrix with a total thickness of 4.4 mm.

At the very end, the shields were modeled and discretized and initial boundary assumptions were adopted. In the middle of the samples, a division of samples in the form of a square with dimensions of 18.38 mm × 18.38 mm was applied in order to locally compact the dimensions of the finite elements. The size of the mesh and the type of finite elements adopted in the middle division for the individual components of the shield material model are presented in the table below (Table 6). In the remaining sample volume, the maximum dimension of finite elements on the outer edges of the samples was 5 mm. The side surfaces of the sample have been restrained, i.e., all translational (Tx = Ty = Tz = 0) and rotational (Rx = Ry = Rz = 0) degrees of freedom have been blocked. Then the linear initial velocity of the projectile was given as 370 m/s, as was the case with the ballistic test. The angular velocity of the projectile was omitted because the literature data indicate that it does not have such a large impact on the simulation results [48,50]. After completion of the aforementioned activities, three sets of simulations were performed for the previously given parameters and types of finite elements. The total simulation time was 150 µs, which allowed the observation of the entire course of the process from impact to complete stop of the projectile. The contact between all elements was set as general contact, and the coefficient of friction *µ* = 0.2 [2,15,19]. The scheme and thickness of individual layers included in the computer geometric model are also presented below (Figure 5).

## 3. Results

The results of the ballistic tests for the prepared samples are presented below and the comparison of obtained results for finite element method simulations for the elements used to model the shields.

### 3.1. Ballistic Tests

**Figure 6:**
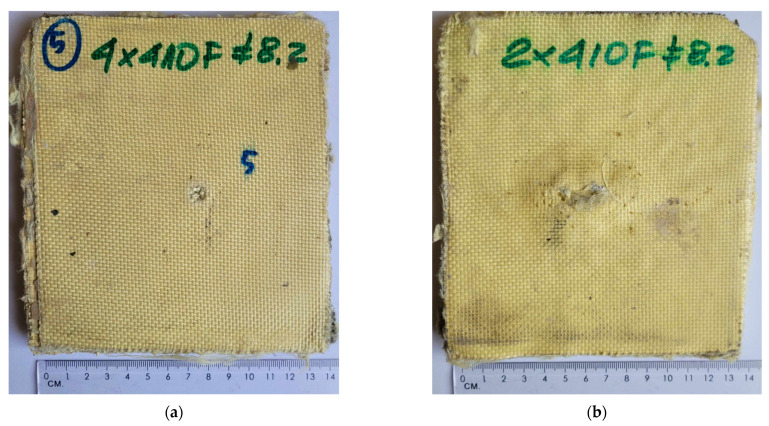
External sample: (**a**) front side; and (**b**) rear side.

**Figure 7:**
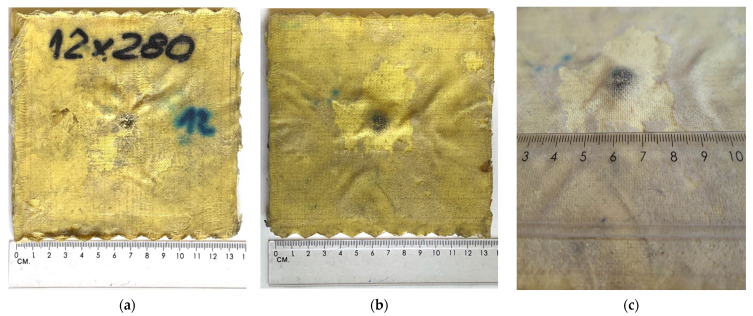
Backing plate sample after shooting: (**a**) front side; (**b**) rear side; and (**c**) rear side at an angle.

Below (Figure 6 and Figure 7), the effects of the ballistic tests can be seen. A full breakthrough was obtained for the external sample consisting of the outer layers of the fabric and filled with a non-Newtonian fluid. A picture of a completely pierced sample is shown below (Figure 6). The impact load of 9 × 19 mm Parabellum ammunition did not completely penetrate the material arrangement of the backing plate sample. On the rear side of the backing sample, a characteristic impact cone for fabric ballistic materials can be seen. The total plastic deflection measured at the highest point of dynamic deformation of the fabric in the backing sample amounted to around 5–6 mm (Figure 7c). The material combination of the samples used did not allow for a complete perforation of the material system of the backing sample.

### 3.2. Numerical Simulations

**Figure 8:**
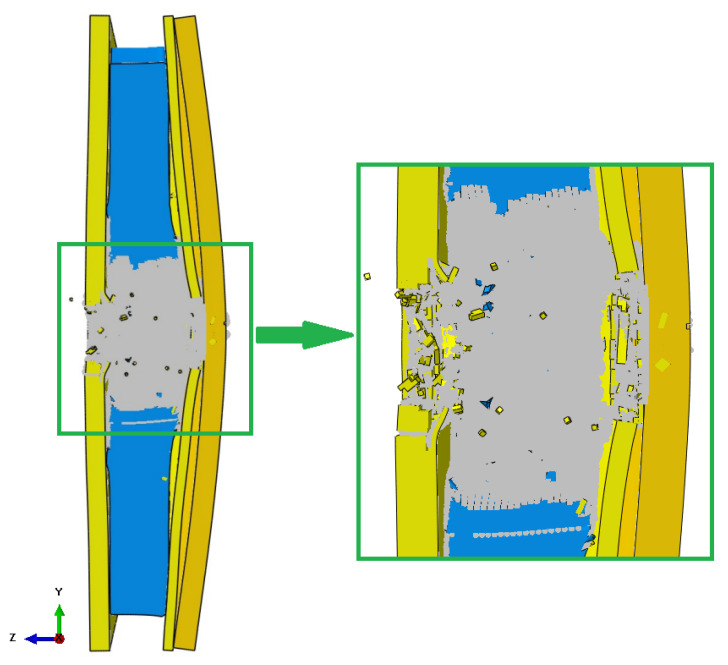
Overall effect of numerical simulations.

This section presents a comparison of the numerical modeling with results obtained experimentally. The general effect of numerical simulations is presented below (Figure 8). As a result of the numerical tests, a complete perforation of the layers made of Twaron^®^ 173 g/m^2^ is noted, as well as a complete puncture of the cells with non-Newtonian fluid. However, the material combination used did not allow for complete penetration of the material system of the sample, which was also obtained during numerical tests. Only the outermost layer of the back sample was damaged, which similarly occurred during the ballistic test. The greatest destruction occurred in the layer with the non-Newtonian fluid. This is, of course, due to the total homogenization that has been carried out in this region. All the unit cells were connected, making the layer homogeneous, with no boundaries between the cells. This caused the shock wave to spread over the entire volume of the layer.

**Figure 9:**
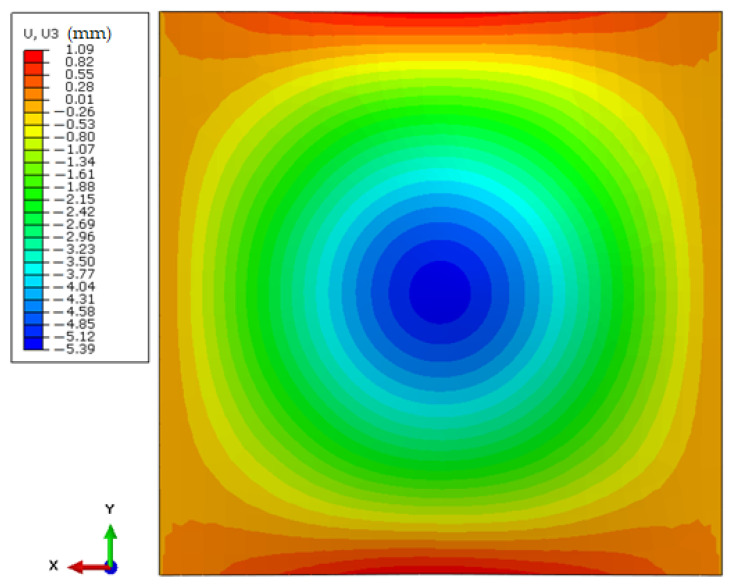
Maximum deflection of the backing plate obtained as a result of numerical simulations—rear side of a backing plate.

As a result of firing the rear sample in previous tests [54], a complete stop of the 9 × 19 mm Parabellum FMJ bullet was obtained; however, the dynamic deflection obtained during the test was then 28 mm. It should be mentioned that the initial speed of the bullet was 350 m/s, while as a result of the current tests it was 370 m/s, and apart from the complete stopping of the bullet, the deflection was also significantly reduced. As a result of the use of additional outer layers and a cartridge with a non-Newtonian fluid, the maximum deflection of the sample was reduced to 5–6 mm, which was presented in Section 3.1. The results obtained during the numerical test were exactly in the same range—the maximum deflection of the backing plate was exactly 5.39 mm as shown in the figure below (Figure 9).

**Figure 10:**
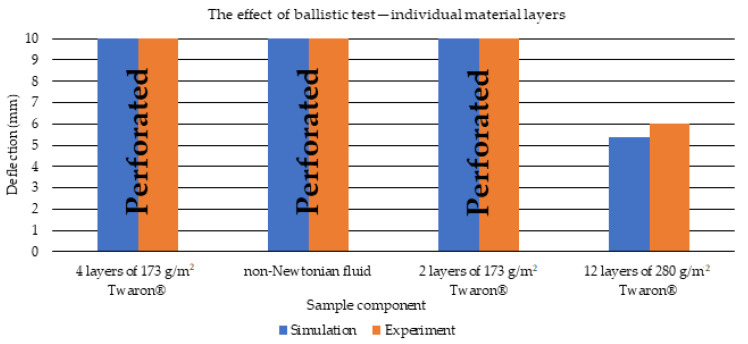
Puncture and deflection of individual material layers of the sample.

Then, a comparison of the obtained puncture of individual layers of the material was presented (Figure 10). As a result of the ballistic test, as in the case of the experiment, the outer sample consisting of 4 layers of 173 g/m^2^ Twaron^®^, non-Newtonian fluid and 2 layers of 173 g/m^2^ Twaron^®^ was completely punctured. However, the backing sample was not completely penetrated. The puncture results of individual layers coincide in the case of simulation and experiment.

The table below (Table 7) presents the maximum values of Mises stress occurring in individual material layers during firing. Although the criterion of failure of the material fibers was not related to the occurring stress, but to the deformation limit of the material at which the failure occurred, the presented values may indicate the correctness of the homogenization and definition of the material. For layers made of Twaron^®^ with worse mechanical properties, much lower stress values were read, and thus material damage occurred at much lower stress values. Since the non-Newtonian fluid is defined by the EOS equation, Mises stress values are not read in this layer.

**Figure 11:**
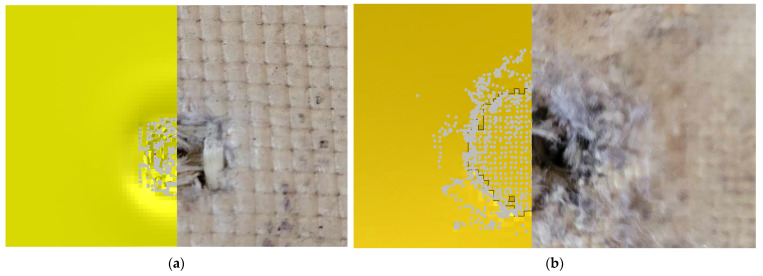
Comparison of obtained material failure after simulation and firing: (**a**) in the outermost layer; and (**b**) in the backing plate.

Below is a comparison of the obtained penetration of the samples at the point of impact of the bullet with fibrous materials. For the outer layer, this effect is shown in (Figure 11a), and for the backing sample in (Figure 11b). The results obtained and the dimensions of the inlet holes are similar to the results obtained as a result of the ballistic test. Possible distortion or destruction of additional elements around the point of impact during the simulation may be affected by the type of finite element (hex) used, as well as its dimension. Gray particles represent elements after conversion—damaged fibers.

**Figure 12:**
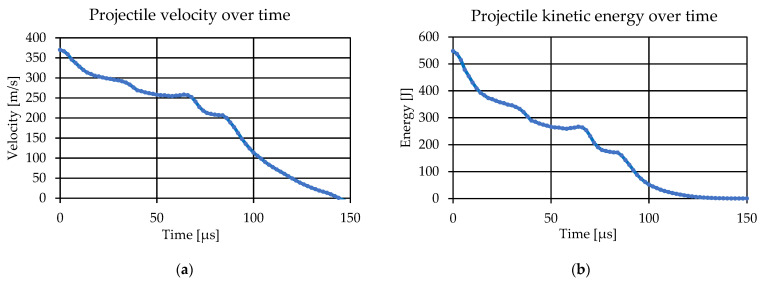
Diagrams obtained as a result of the simulation: (**a**) projectile velocity over time; and (**b**) projectile kinetic energy over time.

Subsequently, the decrease in the speed and kinetic energy of the projectile during the impact was analyzed. In the case of multi-layer, multi-element samples, the analysis of velocity during the perforation of individual armor layers is very difficult, especially during impacts that do not completely penetrate the material arrangement of the samples. Therefore, the simulation is important because of the estimation of the time of penetration of individual layers by the projectile until it stops. Below (Figure 12), the time course of the projectile velocity (Figure 12a) and the time course of the projectile kinetic energy (Figure 12b) are presented. For this purpose, in order to avoid the automatic determination of the kinetic energy for the entire model, 10 points were selected at equal intervals over the entire volume of the projectile, and then the velocity courses over time were made for selected nodes. Subsequently, the data were imported into Excel and the average speed of 10 selected nodes was calculated. Thanks to this, the kinetic energy of the projectile was then calculated. It is noted that the material arrangement of the external sample allowed for a significant reduction in the speed and kinetic energy of the projectile over time, so that during the contact of the projectile with the backing sample a much smaller part of the energy was transmitted to the backing sample, so it also allowed for a significant reduction in deflection compared to previous tests [42,50,54].

**Figure 13:**
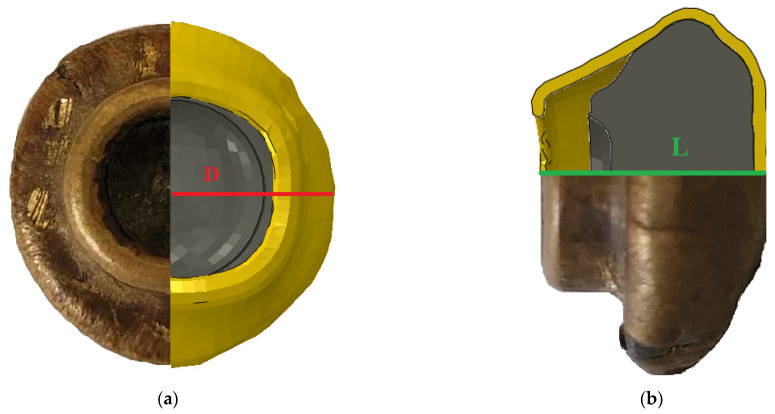
Comparison of projectile deformations after the ballistic tests and numerical tests: (**a**) rear side of projectile; and (**b**) side view of projectile.

The last element to be compared between the real test and the numerical test is the projectile. In order to measure the relative tracking error of the projectile, the length and diameter at the widest point were measured and then compared between ballistic track test and numerical simulations. The results of the measured values are summarized in the table below (Table 8). The deformation observed from the rear is similar for the numerical model to the real projectile, which can be seen in the figure below (Figure 13a). The obtained dimensions of the bullets do not differ much from each other; however, the difference in the shape of the bullets is already noticeable. The side view of the bullet differs to a greater extent, which can be seen in the image (Figure 13b). This state of affairs may be caused by several factors, starting from the adopted model of the constitutive material (this is often influenced by the Johnson–Cook constitutive model, which describes the behavior of the material much worse, especially at very high strain rates compared to other constitutive models [55,56], although more accurate, these models are much less frequently used due to the much greater complexity of determining the material constants) and ending with the size of the finite elements, which can also affect the obtained results.

### 3.3. Comparison of the Obtained Deflection Values to Other Matrices Used in the Literature

**Figure 14:**
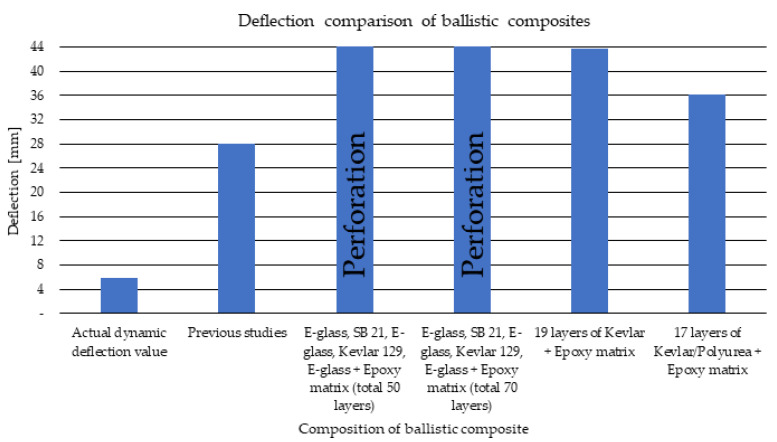
Comparison of obtained deflection value with other studies; previous studies taken from [54], E-glass, SB 21, E-glass, Kevlar 129, E-glass + Epoxy for 50 and 70 layers taken from [57], 19 and 17 layers of Kevlar + Epoxy taken from [58].

So far, no widely used DCPD matrix in ballistic applications has been found in the literature, apart from previous research by the co-authors of this article in the following papers [50,54]. Further development of the methods used to prepare ballistic shields is an element of novelty, thanks to which it is possible to constantly strive to reduce BFS parameters. Below (Figure 14), a summary of the deflection values obtained as a result of the firing of the sample in this study, previous studies and the literature data is presented. As previously mentioned in Section 3.2, BFS was reduced by 22 mm over previous tests by using an external sample containing a non-Newtonian fluid. In research [57], the authors also focused on the production of multi-layer composites that do not contain a non-Newtonian fluid. Many more layers of fabric material were used, the total number of layers was 50 and 70 on the epoxy matrix, and the percentage of fibers in the produced composites was very similar to the content in this article. The average velocity of the projectile was 415 m/s for the 50-layer composite and for the 70-layer composite, 430 m/s. In the next work, the ballistic resistance of Kevlar and Kevlar/polyurea composites on an epoxy matrix was tested at the initial speed of 410 m/s [58]. The use of this type of combination made it possible to meet the assumptions of the NIJ 0101.06 standard, however with a significant limit value of BFS. The use of DCPD resin made it possible to reduce the BFS value in relation to other test results, and the additional use of a sample with a non-Newtonian fluid significantly reduced the BFS value.

## 4. Discussion

The results obtained during the simulations and the ballistic tests are very similar, which indicates that the homogenization process was carried out correctly. The use of a simplified model significantly reduces the time and computational cost of the numerical model. Thanks to the use of the rule of mixtures, a significantly simplified geometric model was obtained, in which there was no need to interlace the fabric or make a representative volume cell. The shortcomings of the method used should be mentioned here. The homogenization process requires equipment, and the method itself is primarily sensitive to the skill and accuracy in separating the fiber material from the matrix and estimating the percentage composition of these components. First of all, it is related to the use of a cutter to cut a fragment from a fibrous sample. Inaccurate execution of the process of cutting out a fragment of the sample may significantly hinder or disturb the accuracy of estimating the percentage composition of fibers and warp. Next, the need to use microscopy to distinguish fibers from resin. In addition, it should also be mentioned that the law of mixtures also assumes many simplifications, such as the perfect connection and saturation of fibers with a matrix, which is also not always successful, and the analysis of the composition only and exclusion at the place of cutting out a fragment of the sample. Nevertheless, this method, with proper attention, allows us to obtain reliable results that are very close to reality, thanks to which it can be treated as a reliable, simplified method for simulating fibers.

One of the main goals of this work was to reduce the dynamic deflection resulting from shelling, which was achieved through the appropriate use of a multi-layer fiber composite with the addition of a non-Newtonian fluid. In the research, the authors proposed the use of DCPD resin, which is not currently used in ballistic applications, and works very well in these applications, as shown by current research. The research target, which was a reduction in dynamic deflection, was achieved. In the analyzed case, the deflection was around 5–6 mm, whereas in previous research it was 28 mm under fire with the same ammunition. The use of an increased percentage of silica allowed to limit the dynamic deflection. Comparing the results of the obtained tests with the literature data, with the applied combination of materials and the innovative DCPD matrix, much better properties of the BFS parameter were obtained in relation to other tests carried out on multi-layer ballistic composites.

## Figures and Tables

**Table 1:** Properties of propylene glycol and the silica powder [43].

Material	*MW* (g/mol)	*ρ* (g/cm^3^)	*T_M_* (°C)	*T_B_* (°C)	*λ* (W/mK)	*η* (Pa·s)
Propylene glycol	76.10	1.036	−59	188	0.34	0.042
Silica	60.08	2.648 (α) 2.196 (amorphous)	1713	2230	-	-

Where: *MW*—molecular weight, *ρ*—density, *T_M_*—melting temperature, *T_B_*—boiling temperature, *λ*—specific heat, *η*—viscosity.

**Table 2:** Material parameters for metallic components of projectile [48].

Material	*E* (GPa)	*v* (-)	*ρ* (kg/m^3^)	*A* (MPa)	*B* (MPa)	*n* (-)	*c* (s^−1^)
Lead	13	0.42	11,300	35	46	0.48	0.01
Brass	130	0.38	8941	112	505	0.42	0.01

Where: *E*—Young’s modulus, *v*—Poisson’s ratio, *ρ*—density, *A*—yield strength at 0 plastic strain, *B*—hardening constant, *n*—hardening exponent, *c*—strain rate constant.

**Table 3:** Material data used before applying the rule of mixtures for fabrics and matrix [44,48,50,51].

Material	*E* (GPa)	*v* (-)	*ρ* (kg/m^3^)	*Re* (MPa)	*G* (GPa)	*FS* (-)
DCPD (dicyclopentadiene)	3.1	0.20	98	-	0.70	0.02
Twaron^®^ 280 g/m^2^	115.0	0.30	1440	3600	3.60	0.10
Twaron^®^ 173 g/m^2^	71.0	0.36	1440	3200	31.00 for *G*_12_ 0.16 for *G*_23_ = *G*_13_	0.10

Where: *E*—Young’s modulus, *v*—Poisson’s ratio, *ρ*—density, *G*—shear modulus, *Re*—yield strength, *FS*—effective fracture strain.

**Table 4:** Material data used after applying the rule of mixtures.

Material	*E*_1_ = *E*_2_ (MPa)	*E*_3_ (Mpa)	*ρ* (kg/m^3^)	*v* (-)	*G* (Gpa)	*Re = Rm* (Mpa)	*FS* (-)
Orthotropic layer for Twaron^®^ 280 g/m^2^	90,735	10,122	1150	0.28	2.99	2840	0.10
Orthotropic layer for Twaron^®^ 173 g/m^2^	56,673	3883	1150	0.33	24.61 for *G*_12_ and *G*_13_ 0.28 for *G*_23_	2525	0.10

Where: *E*—Young’s modulus, *v*—Poisson’s ratio, *ρ*—density, *G*—shear modulus, *Re*—yield strength, *Rm*—maximum strength, *FS*—effective fracture strain.

**Table 5:** Material data used for the non-Newtonian fluid [2,31].

Material	*ρ* (kg/m^3^)	*C*_0_ (km/s)	*s* (-)	*Γ*_0_ (-)
Silica + Polypropylene glycol	2722	2.1	3.75	0.8

Where: *ρ*—density, *s*—Hugoniot slope coefficient, *C*_0_—speed of the sound wave propagating in the material, *Γ*_0_—Grüneisen parameter.

**Table 6:** Mesh and type of finite elements adopted for shield components.

Shield Component	Type	Size (mm)
Layers of 173 g/m^2^ Twaron^®^	Hex	0.5
Non-Newtonian fluid in honeycomb cells	Hex	0.5
Layers of 280 g/m^2^ Twaron^®^	Hex	0.5

**Table 7:** Maximum Mises stress occurring in individual layers of the sample.

Layer	4 Layers of 173 g/m^2^ Twaron^®^	Non-Newtonian Fluid	2 Layers of 173 g/m^2^ Twaron^®^	12 Layers of 280 g/m^2^ Twaron^®^
Mises stress value (MPa)	1300	0	1300	2700

**Table 8:** Comparison of projectile dimensions obtained as a result of the test and simulation.

Projectile	D (mm)	L (mm)
Ballistic test	14.25	8.33
Numerical	14.49	8.53
Relative error	1.70%	2.40%

## Data Availability

Not applicable.

## References

[1] Jamroziak K. (2013). Identification of Material Properties in Terminal Ballistics.

[2] Sen S., Bin Jamal M N., Shaw A., Deb A. (2019). Numerical Investigation of Ballistic Performance of Shear Thickening Fluid (STF)-Kevlar Composite. Int. J. Mech. Sci..

[3] Iannucci L., Dechaene R., Willows M., Degrieck J. (2001). A Failure Model for the Analysis of Thin Woven Glass Composite Structures under Impact Loadings. Comput. Struct..

[4] Zee R.H., Hsieh C.Y. (1993). Energy Loss Partitioning during Ballistic Impact of Polymer Composites. Polym. Compos..

[5] Li S., Sitnikova E. (2020). Representative Volume Elements and Unit Cells.

[6] Pach J., Pyka D., Jamroziak K., Mayer P. (2017). The Experimental and Numerical Analysis of the Ballistic Resistance of Polymer Composites. Compos. Part B Eng..

[7] Tabiei A., Ivanov I. (2004). Materially and Geometrically Non-Linear Woven Composite Micro-Mechanical Model with Failure for Finite Element Simulations. Int. J. Non Linear. Mech..

[8] Soni G., Singh R., Mitra M., Falzon B.G. (2014). Modelling Matrix Damage and Fibre–Matrix Interfacial Decohesion in Composite Laminates via a Multi-Fibre Multi-Layer Representative Volume Element (M2RVE). Int. J. Solids Struct..

[9] Nunes S.G., Scazzosi R., Manes A., Amico S.C., de Amorim Júnior W.F., Giglio M. (2019). Influence of Projectile and Thickness on the Ballistic Behavior of Aramid Composites: Experimental and Numerical Study. Int. J. Impact Eng..

[10] Scazzosi R., Manes A., Giglio M. (2019). An Enhanced Material Model for the Simulation of High-Velocity Impact on Fiber-Reinforced Composites. Procedia Struct. Integr..

[11] Bresciani L.M., Manes A., Ruggiero A., Iannitti G., Giglio M. (2016). Experimental Tests and Numerical Modelling of Ballistic Impacts against Kevlar 29 Plain-Woven Fabrics with an Epoxy Matrix: Macro-Homogeneous and Meso-Heterogeneous Approaches. Compos. Part B Eng..

[12] Briscoe B.J., Motamedi F. (1992). The Ballistic Impact Characteristics of Aramid Fabrics: The Influence of Interface Friction. Wear.

[13] Kirkwood J.E., Kirkwood K.M., Lee Y.S., Ronald G., Egres J.R., Wagner N.J., Wetzel E.D. (2004). Yarn Pull-Out as a Mechanism for Dissipating Ballistic Impact Energy in Kevlar^®^ KM-2 Fabric: Part II: Predicting Ballistic Performance. Text. Res. J..

[14] Duan Y., Keefe M., Bogetti T.A., Powers B. (2006). Finite Element Modeling of Transverse Impact on a Ballistic Fabric. Int. J. Mech. Sci..

[15] Chocron S., Figueroa E., King N., Kirchdoerfer T., Nicholls A.E., Sagebiel E., Weiss C., Freitas C.J. (2010). Modeling and Validation of Full Fabric Targets under Ballistic Impact. Compos. Sci. Technol..

[16] Roylance D., Wang S.S. (1980). Penetration Mechanics of Textile Structures. Methods Phenom..

[17] Das S., Jagan S., Shaw A., Pal A. (2015). Determination of Inter-Yarn Friction and Its Effect on Ballistic Response of Para-Aramid Woven Fabric under Low Velocity Impact. Compos. Struct..

[18] Tan V.B.C.Ã., Shim V.P.W., Zeng X. (2005). Modelling Crimp in Woven Fabrics Subjected to Ballistic Impact. Int. J. Impact Eng..

[19] Nilakantan G., Gillespie J.W.G. (2012). Ballistic Impact Modeling of Woven Fabrics Considering Yarn Strength, Friction, Projectile Impact Location, and Fabric Boundary Condition Effects. Compos. Struct..

[20] Yang E.C., Linforth S., Ngo T., Tran P. (2018). International Journal of Mechanical Sciences Hybrid-Mesh Modelling & Validation of Woven Fabric Subjected to Medium Velocity Impact. Int. J. Mech. Sci..

[21] Nilakantan G., Nutt S. (2014). Effects of Fabric Target Shape and Size on the V 50 Ballistic Impact Response of Soft Body Armor. Compos. Struct..

[22] Nilakantan G., Keefe M., Bogetti T.A., Adkinson R., Gillespie J.W. (2010). On the Finite Element Analysis of Woven Fabric Impact Using Multiscale Modeling Techniques. Int. J. Solids Struct..

[23] Grujicic M., Bell W.C., Arakere G., He T., Xie X., Cheeseman B.A. (2010). Development of a Meso-Scale Material Model for Ballistic Fabric and Its Use in Flexible-Armor Protection Systems. J. Mater. Eng. Perform..

[24] Barauskas R., Abraitiene A. (2007). Computational Analysis of Impact of a Bullet against the Multilayer Fabrics in LS-DYNA. Int. J. Impact Eng..

[25] Otero F., Oller S., Martinez X., Salomón O. (2015). Numerical Homogenization for Composite Materials Analysis. Comparison with Other Micro Mechanical Formulations. Compos. Struct..

[26] Andreassen E., Andreasen C.S. (2014). How to Determine Composite Material Properties Using Numerical Homogenization. Comput. Mater. Sci..

[27] Carvelli V., Poggi C. (2001). A Homogenization Procedure for the Numerical Analysis of Woven Fabric Composites. Compos. Part A Appl. Sci. Manuf..

[28] Szymczak T., Kowalewski Z.L. (2014). Mechanical properties of selected composites and methods of assessing their failure. Transp. Samoch..

[29] Na W., Ahn H., Han S., Harrison P., Park J.K., Jeong E., Yu W.R. (2016). Shear Behavior of a Shear Thickening Fluid-Impregnated Aramid Fabrics at High Shear Rate. Compos. Part B Eng..

[30] He Q., Cao S., Wang Y., Xuan S., Wang P., Gong X. (2018). Impact Resistance of Shear Thickening Fluid/Kevlar Composite Treated with Shear-Stiffening Gel. Compos. Part A Appl. Sci. Manuf..

[31] Petel O.E. (2011). Response of Shear Thickening Materials to Uniaxial Shock Compression. Ph.D. Thesis.

[32] Park Y., Kim Y., Baluch A.H., Kim C.G. (2014). Empirical Study of the High Velocity Impact Energy Absorption Characteristics of Shear Thickening Fluid (STF) Impregnated Kevlar Fabric. Int. J. Impact Eng..

[33] Asija N., Chouhan H., Gebremeskel S.A., Bhatnagar N. (2017). Impact Response of Shear Thickening Fluid (STF) Treated High Strength Polymer Composites—Effect of STF Intercalation Method. Procedia Eng..

[34] Gürgen S., Kuşhan M.C. (2017). The Stab Resistance of Fabrics Impregnated with Shear Thickening Fluids Including Various Particle Size of Additives. Compos. Part A Appl. Sci. Manuf..

[35] Hasanzadeh M., Mottaghitalab V. (2014). The Role of Shear-Thickening Fluids (STFs) in Ballistic and Stab-Resistance Improvement of Flexible Armor. J. Mater. Eng. Perform..

[36] Egres R.G., Lee Y.S., Kirkwood J.E., Kirkwood K.M., Wetzel E.D., Wagner N.J. “Liquid Armor”: Protective Fabrics Utilizing Shear Thickening Fluids. Proceedings of the 4th International Conference of Safety and Protective Fabrics.

[37] Wetzel E.D., Lee Y.S., Egres R.G., Kirkwood K.M., Kirkwood J.E., Wagner N.J. (2004). The Effect of Rheological Parameters on the Ballistic Properties of Shear Thickening Fluid (STF)-Kevlar Composites. AIP Conf. Proc..

[38] Majumdar A., Butola B.S., Srivastava A. (2013). An Analysis of Deformation and Energy Absorption Modes of Shear Thickening Fluid Treated Kevlar Fabrics as Soft Body Armour Materials. Mater. Des..

[39] Cwalina C.D., McCutcheon C.M., Dombrowski R.D., Wagner N.J. (2016). Engineering Enhanced Cut and Puncture Resistance into the Thermal Micrometeoroid Garment (TMG) Using Shear Thickening Fluid (STF)—Armor^™^ Absorber Layers. Compos. Sci. Technol..

[40] Manukonda B.H., Chatterjee V.A., Verma S.K., Bhattacharjee D., Biswas I., Neogi S. (2020). Rheology Based Design of Shear Thickening Fluid for Soft Body Armor Applications. Period. Polytech. Chem. Eng..

[41] Pyka D., Pach J., Jamroziak K. (2019). Numerical Modeling of Ballistic Resistance of Thermoplastic Laminate Under 9 × 19 Mm Parabellum Ammunition. Eng. Mech..

[42] Jamroziak K., Pyka D., Pach J., Bocian M., Kurzawa A., Kurowski J. (2018). Dissipative Properties of Non-Newtonian Fluid under Impact Load. Eng. Mech..

[43] Fürstner A., Ackermann L., Gabor B., Goddard R., Lehmann C.W., Mynott R., Stelzer F., Thiel O.R. (2001). Comparative Investigation of Ruthenium-Based Metathesis Catalysts Bearing N-Heterocyclic Carbene (NHC) Ligands. Chem. Eur. J..

[44] Wisniewski A., Gmitrzuk M. Validation of Numerical Model of the Twaron CT709 Ballistic Fabric. Proceedings of the 27th International Symposium on Ballistics.

[45] (2009). ABAQUS.

[46] Shrot A., Bäker M. (2012). Determination of Johnson–Cook Parameters from Machining Simulations. Comput. Mater. Sci..

[47] Johnson G.R., Cook W.H. (1985). Fracture Characteristics of Three Metals Subjected to Various Strains, Strain Rates, Temperatures and Pressures. Eng. Fract. Mech..

[48] Ziółkowski G., Pach J., Pyka D., Kurzynowski T., Jamroziak K. (2020). X-ray Computed Tomography for the Development of Ballistic Composite. Materials.

[49] Talebi H., Wong S.V., Hamouda A.M.S. (2009). Finite Element Evaluation of Projectile Nose Angle Effects in Ballistic Perforation of High Strength Fabric. Compos. Struct..

[50] Pyka D., Jamroziak K., Ziolkowski G., Pach J., Bocian M. (2018). Research on Ballistic Resistance of DCPD Laminate under Pistol Ammunition Fire. Eng. Mech..

[51] Zhu L., Zhu G., Feng J., Jin L., Ma P. (2019). Impact Tensile Behavior Analysis of Twaron Fiber Tows from Fast Fourier Transform. Text. Res. J..

[52] Małachowski J. (2008). Effect of blast wave on chosen structure—Numerical and experimental study. Int. J. Math. Comput. Simulat..

[53] Małachowski J., Gieleta R. (2008). Security improvement for oil and gas pipeline infrastructure. J. KONES Powertrain Transp..

[54] Bocian M., Pach J., Jamroziak K., Kosobudzki M., Polak S., Pyka D., Kurzawa A., Kurowski J. (2017). Experimental and Numerical Analysis of Aramid Fiber Laminates with DCPD Resin Matrix Subjected to Impact Tests. MATEC Web Conf..

[55] Moćko W., Kowalewski W. (2012). Application of selected constitutive equations to describe the mechanical properties of high-nitrogen steel type VP159. Eng. Model..

[56] Stopel M. (2018). Design of Mechanical Structures Subjected to Loads Increasing at High Speed. Ph.D. Thesis.

[57] Yilmaz H., Bedir F., Elmas U. (2014). Experimentally Investigating the Ballistic Properties of the Polymer-Matrixed Layered and Fiber reinforced Composite Materials. Int. Conf. Appl. Mech. Mech. Eng..

[58] Shih C.H., You J.L., Lee Y.L., Cheng A.Y., Chang C.P., Liu Y.M., Der Ger M. (2022). Design and Ballistic Performance of Hybrid Plates Manufactured from Aramid Composites for Developing Multilayered Armor Systems. Polymers.

